# Qualitative measures that assess functional disability and quality of life in ALS

**DOI:** 10.1186/s12955-022-01919-9

**Published:** 2022-01-21

**Authors:** Susan L. Hartmaier, Thomas Rhodes, Suzanne F. Cook, Courtney Schlusser, Chao Chen, Steve Han, Neta Zach, Venkatesha Murthy, Shreya Davé

**Affiliations:** 1 CERobs Consulting, LLC, Wrightsville Beach, NC USA; 2 Gillings School of Public Health, University of North Carolina-Chapel Hill, Chapel Hill, NC USA; 3 Takeda Development Center Americas, Inc., Cambridge, MA USA

**Keywords:** ALS, ALSFRS-R, Clinical trials, Qualitative outcome measures, HRQoL, ALS cognitive screens

## Abstract

**Background:**

Selection of appropriate trial endpoints and outcome measures is particularly important in rare disease and rapidly progressing disease such as amyotrophic lateral sclerosis (ALS) where the challenges to conducting clinical trials, are substantial: patient and disease heterogeneity, limited understanding of exact disease pathophysiology, and lack of robust and available biomarkers. To address these challenges in ALS, the Amyotrophic Lateral Sclerosis Functional Rating Scale-Revised version (ALSFRS-R) was developed and has become a key primary endpoint in ALS clinical trials to assess functional disability and disease progression, often replacing survival as a primary outcome. However, increased understanding of the ALS disease journey and improvements in assistive technology for ALS patients have exposed issues with the ALSFRS-R, including non-linearity, multidimensionality and floor and ceiling effects that could challenge its continued utility as a primary outcome measure in ALS clinical trials. Recently, other qualitative scale measures of functioning disability have been developed to help address these issues. With this in mind, we conducted a literature search aimed at identifying both established and promising new measures for potential use in clinical trials.

**Methods:**

We searched PubMed, Google, Google Scholar, and the reference sections of key studies to identify papers that discussed qualitative measures of functional status for potential use in ALS studies. We also searched clinicaltrials.gov to identify functional status and health-related quality of life (HRQoL) measures that have been used in ALS interventional studies.

**Results:**

In addition to the ALSFRS-R, we identified several newer qualitative scales including ALSFRS-EX, ALS-MITOS, CNS-BFS, DALS-15, MND-DS, and ROADS. Strengths and limitations of each measure were identified and discussed, along with their potential to act as a primary or secondary outcome to assess patient functional status in ALS clinical trials.

**Conclusion:**

This paper serves as a reference guide for researchers deciding which qualitative measures to use as endpoints in their ALS clinical trials to assess functional status. This paper also discusses the importance of including ALS HRQoL and ALS cognitive screens in future clinical trials to assess the value of a new ALS therapy more comprehensively.

## Background

Amyotrophic lateral sclerosis (ALS) is a progressive neurodegenerative disorder of motor neurons characterized by loss of physical function across various domains (bulbar, arm/leg motor, and respiratory) and average survival of 3–5 years from symptom onset [1]. Given the poor prognosis and dearth of effective treatments, development of new therapeutic approaches is of primary importance for ALS patients.

Historically, the primary endpoint in ALS clinical trials was survival, defined as survival or tracheostomy, necessitating relatively long trial duration particularly in patients with less severe ALS, and imposing difficulties associated with personal preference with regards to end-of-life choices and access to assistive technology and tracheostomy impacting trial outcome. Objective measures such as quantitative muscle testing and handheld dynamometry to assess muscle strength, and vital capacity (VC) to assess ventilatory muscle strength were used to assess ALS functional status. Early clinical ALS rating scales such as the Norris Scale [2] and the Appel Scale [3] combined interview and objective functional assessments. All these methods were lengthy, required clinician time and specialized equipment to administer, and were not feasible if patients were too ill to visit a medical clinic.

In response, the Amyotrophic Lateral Sclerosis Functional Rating Scale (ALSFRS) and its revised form Amyotrophic Lateral Sclerosis Functional Rating Scale-Revised (ALSFRS-R) was developed in the 1990s as a qualitative measure of function to evaluate the clinical characteristics of ALS [4, 5]. Since then, the ALSFRS (and later the ALSFRS-R) has become the most widely applied rating scale in ALS in clinical trials as a primary or secondary outcome and is considered the gold standard measure of functional disability and disease progression in ALS patients. It is an accepted primary endpoint measure for Phase 3 ALS clinical trials to monitor functional decline patients over time [6–9] and recommended as part of the EMA and FDA Guidance for ALS drug development [8, 9], although survival is still often measured as a secondary endpoint and EMA considers it a critical part of assessment of efficacy [8].

### Why look at other qualitative measures to assess ALS functional disability?

Almost 30 years after development, the ALSFRS-R is sometimes criticized as being too rudimentary to accurately track disease progression [10]. Reports vary regarding the linearity of the measure over time, with early and late phases of ALS showing the quickest rates of decline [11–14], while heterogeneity of the disease may affect ALSFRS-R results between ALS clinical subgroups [12, 15, 16].

Rasch analyses of the ALSFRS-R have demonstrated its lack of unidimensionality, meaning it better constitutes a profile of domain scores than a total overall score of disease severity. Rasch analyses also supports three domains instead of four, recommending the collapse of fine and gross motor domains into one, and prescribes a better fit with a 0–2 response instead of 0–4 [17–20]. Substantial misfit of many ALSFRS-R items, including overlapping response options and disordered thresholds indicating issues with patients’ ability to discriminate between items have been reported, with only the items on the bulbar domain showing good fit [20].

Grade response monitoring (GRM) analysis [19] of the ALSFRS-R using the largest publicly available repository of merged ALS clinical trials data (PRO-ACT; https://nctu.partners.org/ProACT/Data [21]) indicated floor effects (poor discrimination in more severe patients) for the items “dressing and hygiene” and “climbing stairs” on the gross motor domain and ceiling effects (poor discrimination in patients with milder disability) for the items “speech”, “salivation”, and “swallowing” on the bulbar domain and all items on the respiratory domain [19, 21]. This suggests the ALSFRS-R may not adequately assess ALS patients with more severe motor disability, less severe bulbar disability or lesser respiratory severity (or that patients upon first clinical trial visit may self-select or be selected for having minimal respiratory dysfunction) (see Table 1 for the full analysis [19]). GRM analysis [19] does support the ALSFRS-R having 4 domains, although revision to some of the items and the response options is recommended to help clarify their meaning.

**Table 1:** Items on the ALSFRS-R that did not discriminate well as identified by GRM [19]

Domain item	Response options that do not discriminate well
Bulbar domain Salivation	Time 1 R1 ‘marked excess of saliva with some drooling’ vs R0 ‘marked drooling; requires constant tissue or handkerchief’ R2 ‘moderately excessive saliva; may have minimal drooling’
Bulbar domain Swallowing	Time 0 R1 ‘needs supplemental tube feeding vs R0 ‘nothing by mouth; exclusively parenteral or enteral’ R2 ‘dietary consistency changes’
Fine Motor domain Cutting food and handling utensils	All times 0, 1, 2 All responses far exceeded the threshold for acceptable item discrimination This item may potentially be redundant May be over discriminating between individuals with different levels of severity as assessed by this item
Fine Motor domain Dressing and hygiene	Time 0 All responses exceeded the threshold for acceptable item discrimination
Gross Motor domain Turning in bed and adjusting bed clothes	Time 0 R0 ‘helpless’ vs R1 ‘can initiate but cannot adjust sheets alone’
Gross Motor domain Climbing stairs	All times 0, 1, 2 R2 ‘mild unsteadiness or fatigue’ vs R1 ‘needs assistance’ R3 ‘ slow’ *Most problematic item*
Respiratory domain Respiratory insufficiency	At all times 0, 1, 2 Patients responded with either 4 ‘none’ or 2 ‘continuous use of BiPAP’ rather than 3 ‘intermittent use of BiPAP, suggesting that response 3 did not assess a unique level of severity
Respiratory domain Orthopnea Respiratory insufficiency	Fit very poor for these items and lowest threshold ‘0’ could not be estimated due to lack of responses in that score category

GRM: Grade Response Modeling, [Source: Bacci 2016], [19]

These studies suggest that the ALSFRS-R, in its current form may not be the “best” as a single primary outcome measure to track ALS disease progression in a clinical trial. Revising the ALSFRS-R to address some of the issues discussed above could improve its performance; however, any modification will require additional validation of the modified ALSFRS-R measure in accordance with current FDA guidelines for PRO development [22] and in line with the FDA guidelines for ALS [9] suggesting that ALSFRS-R should be supplemented with additional functional measures. Recently developed qualitative functioning scales that have addressed these issues could offer an alternative to the ALSFRS-R, either as a primary outcome or as a supplemental measure to the ALSFRS-R to assess functional disability in clinical trials.

These identified issues with the ALSFRS-R, including non-linearity (potentially leading to incorrect statistical assumptions and spurious associations with the rate of decline) [11], multidimensionality (in that it better constitutes a profile of domain scores than a total overall score representing disease severity) [15, 17, 18] and floor (poor discrimination in more severe patients) and ceiling (poor discrimination in patients with milder disability) effects [19], have challenged its continued utility as a primary outcome measure in ALS clinical trials and driven the development of other qualitative scale measures of functional disability in ALS.

This paper serves as a reference guide for researchers deciding which qualitative measures to use as endpoints in their ALS clinical trials to assess functional status. It provides a targeted overview of the ALSFRS-R and newer qualitative scales along with their potential to act as primary or secondary outcomes in ALS clinical trials. It has also been suggested that different, or at least complementary ways to assess the value of a new therapy would be to include measures of the treatment’s impact on quality of life (QoL) [23, 24] along with cognitive screening measures [25].

With this in mind, we present the results of a search of published and grey literature aimed at identifying both established and promising new measures for potential use in clinical trials. To our knowledge, no papers exist that provide a collected list such as this. This paper further discusses the utility of including ALS health-related quality of life (HRQoL) measures and ALS cognitive screening measures in future clinical trials to more fully assess the patient perceived value of a new therapy and to help determine if cognitive or behavioral impairment has an impact on physical or motor functioning, particularly in more severe or elderly ALS patients.

## Methods

Two researchers searched PubMed, Google, Google Scholar, and the reference sections of key studies to identify papers that discussed qualitative measures of functional status for potential use in ALS studies. Qualitative or subjective measures of ALS functional status are explored as opposed to objective measures such as muscle strength, muscle electromyography, vital capacity, walking tests and include disease specific and general instruments. An initial search used terms associated with ‘amyotrophic lateral sclerosis’, ‘qualitative measure’, ‘functional status’, and ‘patient-reported outcomes’. Studies were included if they were English-language and were published from January 2000 through April 2021. Titles and abstracts were first screened by one reviewer to determine whether the study provided information on qualitative measures for ALS functioning. Full-text reviews were then conducted in cooperation between the two researchers to extract relevant information on the use of the measure including strengths and limitations. The two researchers discussed their findings together and agreed which papers were relevant to the research. Data on the measure content, validity, use in published literature, and any noted strengths or weaknesses were extracted in cooperation by two researchers and discussed with all authors.

We also searched clinicaltrials.gov to identify any additional scales that had been used in clinical trials to measure functional ability or quality of life in patients with ALS using keywords such as “ALS”, “scales”, and “functional measures”. Studies had to be registered, industry-sponsored, Phase 2–4, interventional with ‘Not yet recruiting’, ‘Recruiting’, ‘Active/not recruiting’, or ‘Completed’ status. Data on the phase, primary, secondary, and exploratory measures, sponsor, and status were extracted by a single reviewer and discussed with a second researcher.

## Results

### Qualitative assessment of functional decline in ALS clinical trials

#### ALSFRS-R

The ALSFRS-R is well validated, reliable, simple, brief, and requires no equipment or special training. It can be completed by the clinician, patient self-report, or proxy caretaker for those with more severe disease [7]. It has 12 items and assesses current disability across 4 domains—bulbar, fine motor, gross motor, and respiratory. Each item has five ordinal response options ranging from 0 (loss of function) to 4 (normal function), with a total score ranging from 0 to 48; higher scores indicating a higher level of functioning.

Developed with clinician input, initial validity was established by documenting that change in ALSFRS scores correlated with change in strength over time, measured by isometric muscle strength (r = 0.60 with fine motor and gross motor domain), and lung function [forced vital capacity (FVC) r = 0.55 with respiratory domain], while total ALSFRS-R baseline scores strongly predicted survival across 9 months in ALS patients (HR: 0.94) [4, 26]. The ALSFRS-R added additional assessments of respiratory dysfunction, including orthopnea, and need for ventilatory support, making the respiratory function equal in weight on overall score to other domains such as fine and gross motor function [5], but retained properties of the original scale, showing strong internal consistency [intraclass correlation (ICC) 0.73 total score], interrater reliability (0.93–0.95) and construct validity with survival, death/tracheostomy [5, 7, 12, 27–29], length of hospital stay and survival in ALS patients with acute respiratory failure on mechanical ventilation [30].

The ALSFRS-R was validated for self-administration [31], can be performed in person or via telephone [32], smartphone [33], and videoconference [34], making it particularly useful when patients are unable to attend clinic.

Current ALS clinical trials (from clinicaltrials.gov) often define a clinical response as a rate or slope of change of ALSFRS-R over time. On average, patients with ALS in the community have a decline of an average of -1 point/month [7], but clinical trial populations vary based on inclusion criteria [21]. For example, recently released Phase II results for the ALS CENTAUR trial for AMX0035 reported ALSFRS-R scores for the treated group declined less than the placebo group (1.24 vs. 1.66 points per month) [35]. On the other hand, in the PRO-ACT database of completed ALS clinical trials, average progression was a decline of 0.7 point/month [21]. A survey of 65 clinicians of the Northeast ALS Consortium (NEALS) reported that the majority of clinicians and clinical researchers surveyed believed that a therapy that resulted in a change of 20% or greater in the slope of the ALSFRS-R would be the percentage in which a somewhat clinically significant change starts to be noted [13].

### New qualitative measures for the assessment of functional disability in ALS clinical trials

#### ALS functional rating scale extension (ALSFRS-EX)

Improvements in assistive technology have led to increased survival in ALS, with patients experiencing continued changes in their physical functioning despite having reached the lower bounds of the ALSFRS-R (floor effects). As a result, an online community for people with ALS (PALS) were recruited to construct and pilot new items to add to the ALSFRS-R scale to improve its sensitivity at lower levels of physical function in patients with advanced ALS. Item generation and item reduction processes led to the addition of 3 new items to the ALSFRS-R, (1) ability to use fingers to manipulate devices (fine motor), (2) ability to show emotional expression in the face (bulbar), and (3) ability to get around inside the home (gross motor). Additional items used the same 5-point ordinal scale as the ALSFRS-R where a score of 0 represents a total loss of function and 4 represents normal function [36].

The overall original ALSFRS-R scale scores and extended scale scores were correlated 0.99 over the 1-week re-test. The ALSFRS-EX was able to detect a 3-month change in a group of 20 ALS patients with the lowest functional status (0–12), whereas the original ALSFRS-R did not (t = 2.727 vs t = 1.395) [36].

Additional validation studies in ALS clinical trials (i.e., longitudinal validation in ALS clinical trial populations) are required to assess the utility of the ALSFRS-EX as a possible replacement for the ALSFRS-R in clinical trials, particularly in patients with more advanced disease.

#### ALS Milano–Torino staging (ALS-MITOS)

The ALS-MITOS staging system [37] was proposed as a novel “one scale measures all” tool to measure the progression of ALS and its ability to serve as a proxy for long-term survival. It was thought that a valid staging system should correlate with ALS disease progression, as well as quality of life QoL and economic burden, and can be derived from the ALSFRS-R. The ALS-MITOS includes 6 stages based on functional ability, based on the 4 key domains from the ALSFRS-R (walking/self-care, swallowing, communicating and breathing). Each domain has a threshold reflecting the loss of function in the specific ALSFRS-R subscores. Values of 0 (below threshold) or 1 (above threshold) are assigned, and the stages are determined as the sum of values across the four domains. Six stages are defined: stage 0 indicates no loss of function in any domain; stages 1–4 represent the loss of independence of function in 1, 2, 3 or 4 domains, and stage five is death. A similar staging system, King’s Staging, is also frequently used, but is not a solely qualitative measure, as it included quantitative assessments. [38]

Studies showed ALS patients progressed to higher stages of disease at 12 months compared with their baseline stage; functional (ALSFRS) and QOL measures were inversely related to disease stage, and health service costs were directly and significantly related to increasing disease stages from 0 to 4 [37, 39]. ALS progression from baseline to 6 months as defined by the ALS-MITOS system predicted death, tracheostomy or > 23-h non-invasive ventilation (NIV) [40]. The ALS-MITOS developers suggest the staging system can reliably predict the course of ALS up to 18 months and can be considered a novel and valid outcome measure in ALS clinical trials; however additional validation studies are required, particularly longitudinal validation in a clinical trial.

### Center for neurologic study bulbar function scale (CNS-BFS)

Dysphagia occurs in about 85% of patients at some point during the ALS process and is associated with malnutrition, weight loss, reduced QOL, aspiration pneumonia, and death [41–43]. Early detection and consistent monitoring of dysphagia provides the opportunity to improve survival and QOL with timely interventions. The ALSFRS-R has reported poor discrimination in patients with milder disability for the items, “speech”, “salivation”, and “swallowing” on the bulbar domain [19] as well as inadequate diagnostic accuracy of the swallowing item to detect radiographically confirmed swallowing impairment, suggesting the need for alternate measures to assess dysphagia in ALS [44].

The CNS-BFS is a 5-min, 21-question self-administered questionnaire that assesses three domains of bulbar function: speech, swallowing, and salivation. Recall is one week and for each domain, subjects are asked to rate seven items on a scale of 1 (does not apply)–5 (applies all of the time). Scores range from 21 (no symptoms of bulbar dysfunction) to 112. Internal consistency was 0.97, and all three domains were highly correlated with the Global General Impression Scale (r = 0.83 to 0.95) [32] and test–retest reliability over a 2-week screening interval was 0.86. The CNS-BFS total score and ALSFRS-R bulbar subscale were highly predictive of clinician diagnosis of impaired bulbar function [receiver operating characteristic (ROC) area under the curve (AUC), 0.95 and 0.92, respectively] and the CNS-BFS total score was highly and significantly correlated with the bulbar subscale of the ALSFRS-R (r = − 0.90) [45].

When compared to the composite ALSFRS-R, the speech domains of both the CNS-BFS and the ALSFRS-R bulbar scale were sensitive measures of a treatment effect [45]. In contrast, the swallowing and salivation domains of the CNS-BFS were both responsive to treatment (whereas the swallowing and salivation questions on the ALSFRS-R were not. Each of these associations was statistically significant [45].

#### Dyspnea ALS scale (DALS-15)

Dyspnea occurs in about 80% of ALS patients during the course of disease [46]. The DALS-15 [47, 48] is a 15-item, ALS-specific self-reported questionnaire developed with Rasch methodology to detect and quantify dyspnea. Recall is the past two weeks and response options are never (0), occasionally (1), and often (2). Item thresholds are distributed across the entire spectrum of dyspnea and not clustered, so dyspnea can be estimated with good accuracy over a wide range without a ceiling or floor effect. The sum score can be easily computed by summing the individual item scores to obtain an overall score ranging from 0 to 30 points. Cronbach’s alpha was 0.88. Test–retest reliability was 0.98. Minimally detectable change was 3.21 (10.87%) on the 0–30 scale. The DALS-15 correlated highly with the respiratory subscale of ALSFRS-EX (r = − 0.56), Borg scales (r − 0.52, 0.50) and the SRI (severe respiratory insufficiency) subscale of respiratory complaints (r = − 0.75). The scale was able to detect significant differences between patients with and without NIV. The DALS-15 is considered most valuable for the guidance of patients in later stages when NIV is already introduced, and in patients with severe bulbar dysfunction in whom assessment of respiratory function is difficult due to loss of speech and inability to perform spirometric tests.

#### Motor neuron disease—dyspnea scale (MND-DS)

The newly developed MND-DS may be a valuable tool for remotely monitoring respiratory function between clinic visits in patients with motor neuron disease (MND) including ALS. Developed in accordance with the FDA 2009 guidance for patient-reported outcomes (PROs) [22], the MND-DS has three self-reported dyspnea symptoms (1) dyspnea while eating/talking, (2) dyspnea while lying flat, and (3) dyspnea during light activity [49]. Each item is scored from 0 to 4, resulting in a possible total score between 0 (no dyspnea) and 12 (severe dyspnea), with an optimal cut-off-score of ≥ 2 with 75% sensitivity. Significant correlation with the ALSFRS-R respiratory domain was observed at 0.6, reliability was adequate with ICC values ranging from 0.66 to 0.90 and the scale was responsive to disease severity with higher MND-DS scores in patients with more severe dyspnea [49]. The MND-DS showed better diagnostic performance than the ALSFRS-R respiratory domain [49], suggesting that the MND-DS may be a preferred option to identify patients with a reduced respiratory function upon entry into clinical trials or as a supplemental measure to the ALSFRS-R in clinical trials where treatment is expected to have the largest impact on respiratory functioning. Validation studies will need to be conducted before the MND-DS can be considered as a key outcome measure in ALS clinical trials.

#### Rasch overall ALS disability scale (ROADS)

Using Rasch methodology and measure development in accordance with FDA 2009 guidance for PROs and the 2019 FDA guideline for ALS drug development [9], the recently developed ALS disability scale, ROADS, is a 28-item, self-reported questionnaire targeting a broad range of disability levels expected to be more responsive in detecting clinical changes than the ALSFRS-R. Each item is scored as 0 (unable to perform), 1 Abnormal (able to perform but with difficulty compared to before ALS symptoms) or 2 Normal (able to perform without difficulty as before ALS symptoms).

Test–retest reliability for the ROADS was good (ICC = 0.97), construct validity was good with the ALSFRS-R (r = 0.82) and moderate with vital capacity percentage (r = 0.57). ROADS variance explained by the measured construct was 58.2%, considered sufficient for unidimensionality [50]. The ROADS is linearly weighted, meaning that a 1-point change in the overall normed score captures a measurable unit of disability that is consistent across the entire scale, and 2-point changes reflect twice the disability level compared with a 1-point change. The ROADS developers suggest it provides a more consistent and sensitive grading scale than the ALSFRS-R, allowing for better tracking of ALS disease progression. Future studies of the ROADS should examine its longitudinal performance, assess correlation of ROADS with survival, and examine predictive features of the scale. Ongoing studies are also planned to determine test–retest reliability for telephone-administered scales and scales completed by live-in caregivers [51].

Table 2 provides a summary comparison of the measurement properties among the qualitative measurement scales for functioning. Table 3 describes the strengths and weaknesses of the qualitative scales that assess functional disability.

**Table 2:** Summary comparison of measurement properties among ALSFRS-R and more recent qualitative measurement scales for functioning

	ALSFRS-R	ALSFRS-EX	ALS-MITOS^a^	CNS-BFS	DALS-15	MND-DS	ROADS
*Conceptual model*							
Construct defined	✓	✓	✓	✓	✓	✓	✓
Target population defined	✓	✓	✓	✓	✓	✓	✓
Expected subscales described	✓	✓	✓	n/a	n/a	n/a	n/a
*Content validity*							
Patient Input	X	✓	^a^	✓	✓	✓	✓
Expert Input	✓	✓	✓	✓	✓	✓	✓
Description of item development (Item generation /reduction)	X	✓	^a^	✓	✓	✓	✓
*Reliability*							
Test retest	✓	✓	^a^	✓	✓	✓	✓
Internal consistency	✓	✓	^a^	✓	✓	✓	✓
*Construct validity*							
Convergent	✓	✓	✓	✓	✓	✓	✓
Longitudinal	✓	✓	✓	✓	In progress	TBD	In progress
*Responsiveness*							
Across disease subgroups	✓	✓	✓	✓	✓	✓	✓
Functional status	✓	✓	✓	✓	✓	✓	✓
Therapy/treatment	✓	TBD	TBD	✓	In progress	TBD	In progress
*Interpretation and scoring*							
Plan for scoring measure	✓	✓	✓	✓	✓	✓	✓
Scaling described	✓	✓	✓	✓	✓	✓	✓
*Ease of use/patient burden*							
Easy to administer	✓	✓	✓	✓	✓	✓	✓
Length reasonable—minimal patient burden	✓	✓	✓	✓	✓	✓	✓

*TBD* to be determined

^a^Staging system developed as a novel way to interpret the scoring of the already validated ALSFRS-R domains. No item generation or reduction required. As such, no patient input sought. No additional clinical expert input sought

**Table 3:** Strengths and limitations of ALSFRS-R and other qualitative scale measures that assess functional disability

Tool	Description	Strengths	Limitations	Recommendations for best use in a clinical trial
ALSFRS-R [7] https://alspathways.com/hcp/why-alsfrs-r-matters/ https://www.neurotoolkit.com/alsfrs-r/	12-item self-reported function scale of bulbar, fine motor, gross motor, respiratory domains	Patient centered Fast, easy administration (clinician, self or proxy, in person or via telephone) Cost efficient Long history of reliability and validity Validated as a predictor of survival Frequently used Supported by EMA and FDA Translated into many languages with cultural adaptations	Little information on its development and patients Not responsive to progression of disease if non-linear Multidimensional; total score may not be meaningful if not stratified on type of ALS onset Floor and ceiling effects, unable to capture early or late-stage clinical changes Use of ordinal raw scores, 1-point changes can represent a small or large loss of functional ability depending on the domain Lack of discrimination between response categories can lead to response variability	If used as primary outcome, FDA and EMA require supplemental objective primary outcome of function Supplement with a newer function measure (e.g., ROADS) Consider domain subscores with total score Use Rasch or GRM methodology to account for non-linearity of ALS progression; stratify on tyle of onset Supplement with function or symptom specific measure to discriminate more severe and less severe patients Stratify on predictors of functional decline
ALSFRS-EX [36]	Addition of 3 items to the 12-item ALSFRS-R (2 motor, 1 bulbar)	Based on an already validated measure New items based on PRO guidelines Sensitive to change in low-functioning patients	Limited validity data	Could be substituted for the ALSFRS-R for improved sensitivity in patients with advanced ALS, on assisted technology and lower levels of functioning
ALS-MITOS [37]	Based on domains of the ALSFRS-R. Focuses on the rate of loss of each function within each domain	Based on already validated measure Considers lost functions within each domain Sensitive to smaller treatment effect Stages correlate with generic QoL (SF-36) and health service costs	Limited use data Further investigation of transitions through stages required to further assess the utility of this proposed staging system	Supplement to the ALSFRS-R, particularly when additional measures of QOL or health care costs are not feasible
CNS-BFS [45]	A 21-item self-administered scale across bulbar functioning—speech, swallowing, salivation	Swallowing and salivation items appear more sensitive to treatment effect than in the ALSFRS-R Developed using FDA PRO guidelines	Bulbar specific – would need other measures to assess treatment impact on other functions	Supplement to the ALSFRS-R as a more sensitive measure of bulbar dysfunction Use as primary or key secondary outcome measure if treatment is aimed at reducing bulbar decline
DALS-15 [47]	Self-reported 15 item Rasch modeled scale to assess dyspnea in ALS	Follows FDA PRO guidelines Satisfies Rasch model with good fit Optimal targeting Unidimensional, meaningful overall score	Dyspnea specific—needs other measures to assess treatment impact on other functions Limited validity data	Use to identify ALS patients with dyspnea Aid in the early assessment and monitoring of dyspnea for symptom management as an outcome measure supplementary to the ALSFRS-R respiratory domain
MND-DS [49]	Self-reported 3 item measure of dyspnea in MND patients including ALS	Follows FDA PRO guidelines Better diagnostic performance for capturing reduced respiratory function than the ALSFRS-R respiratory domain	Limited validity data. No longitudinal validity data Supine vital capacity used to assess respiratory functioning instead of the gold standard (trans-diaphragmatic pressure) Assessments across MND centers not standardized	Consider use as secondary outcome or as a supplement to the ALSFRS-R respiratory domain if treatment is aimed at reducing respiratory decline Use to monitor respiratory functioning remotely, between site visits, between pulmonary function testing during a clinical trial or in patients unable to travel Use to identify/screen patients with reduced respiratory functioning
ROADS [20]	Self-reported 28-item Rasch modeled ALS disability scale	Follows FDA PRO guidelines Satisfies Rasch model with good fit Linearly weighted Unidimensional, meaningful total score Targets broad range of disability levels May be more sensitive to smaller changes in functioning	New measure with limited validity data Longitudinal validity currently not available but is being assessed in ongoing clinical trial(s) No translations or cultural adaptations available	Potential replacement for the ALSFRS-R Supplement to the ALSFRS-R until longitudinal validity data is available

### Assessment of HRQoL in ALS clinical trials

HRQoL is a key measure that should be considered as a key outcome along with functional status. This is even more important given that ALS patients are surviving longer than before [24]. Table 4 describes the strengths and weaknesses of the more commonly used qualitative scales that assess HRQoL in patients with ALS.

**Table 4:** Quality of life measures and cognitive screening measures in ALS

Tool	Description	Strengths	Limitations	Recommendations for best use in a clinical trial
ALSAQ-40 [52, 53]	40-item disease specific HRQoL. Many items similar to ALSFRS-R	A validated ALS-specific HRQoL measure developed in accordance with FDA PRO guidance	40 items may be too impractical for use in a clinical trial due to increased patient burden	Use as secondary endpoint Use in addition to a function measure to provide extra sensitivity in more severe disease
ALSAQ-5 [54]	5-item measure generated from the ALSAQ-40	Provides a quick measure of HRQoL with minimal patient burden	Shorter instrument lacks measurement precision of larger measures	Use when HRQoL is an exploratory endpoint May be a valid alternative to the ALSAQ-40 (i.e., reduced patient burden, costs)
ALSSQOL-20 (ALSSQOL-SF) [55]	20-item disease specific measure of global QOL reduced from the larger ALSQOL	Short administration time suitable for clinical use	Use is better in clinical care Focus is more on global QoL than HRQoL Only 2 bulbar items	Use in an interventional study to determine treatment effect *if* combined with an HRQoL measure
EQ-5D Self-report [56–58]	5-items on mobility, self-care, usual activities, pain/discomfort, anxiety/depression plus a self-report of current health	Quick, easy to complete Well validated measure very commonly used in clinical trials	Quick snapshot of patient’s current state of health but *not specific to ALS*	Use when a health economics outcome is required or if general HRQoL is a secondary or exploratory endpoint
WHOQOL-BREF [59]	Recently validated, self-reported 26-item generic measure of QoL. Domains include physical health, social relationships, and environment	Quick, easy to complete High compliance reported in ALS Available in 19 different languages High reliability and construct validity Enables comparisons between ALS conditions and general population Interval level measurement (Rasch)	Social domain showed an unsatisfactory fit to the Rasch model Generic measure, may not capture QoL items specific to ALS patients Limited use data in ALS patients Limited longitudinal validity data	Use for parametric analysis and for comparison with other conditions or general populations
Neuro-QoL [24]	Short form measures of functioning, ADLS Self/proxy report, 6–8 items	Very well developed and validated function and ADL measures (FDA PRO guidance) in a population of patients with neurodegenerative diseases including ALS	Not feasible to include all the individual measures to assess overall functioning Limited validity data in ALS specific populations	Another HRQoL option to the ALSAQ-20 Select the appropriate function / symptom measure for highest treatment impact Secondary or exploratory measure of HRQOL to supplement a primary function measure
PROMIS-10 Global Health [24, 60]	Self-reported short form generic Global Health measure of mental and physical health	Well developed and validated Simple, easy to administer Provides mental and physical health scores	May be too general for use in an ALS population when other disease specific HRQoL measures exist Limited validity data in ALS specific populations	Use for quick global measure of HRQoL rather than disease specific for ALS (secondary or exploratory measure) Can be used in collaboration with NeuroQoL measures (fatigue)
ALS Cognitive Behavior Screen (ALS-CBS) [63–65]	Clinician assessed, 10-item cognitive section and 18-item, caregiver-rated behavioral section to identify patients who have cognitive or behavioral changes suggestive of frontal temporal dementia (FTD)	Validated against neuropsychological tests Can distinguish between cognitively impaired and non-impaired patients Accommodates decline in motor functioning Caregiver/self-reported behavior section Administration time < 10 min	Requires health professional for assessment of cognitive section which incurs added expense Test–retest not established Does not assess language or social cognition Behavioral component may not capture mild behavioral change	Use as initial screening of cognitive or behavioral changes within the population that might affect assessment of disease severity and progression and potentially confound response to therapy
Edinburgh Cognitive and Behavioral ALS Screen (ECAS) [61, 66–68]	ECAS-cognitive screen comprises 16 items divided into ALS-specific subscale and a non-ALS-specific subscale. ECAS-behavioral screen includes 5 domains of behavior	Validated against a battery of neuropsychological tests Can provide early identification of cognitive and behavioral changes Accommodates decline in motor functioning Assesses language and social cognition, may be suitable for patients with bulbar disability	Guidelines prefer that a neuropsychologist administer or supervise ECAS administration (added expense) 45 min + administration time Behavioral component requires interviewing the caregiver May not be feasible in clinical trials due to increased patient burden	Use for screening cognitive or behavioral changes that might affect assessment of ALS disease severity and progression and potentially confound response to therapy Use when decline in functioning in more severe ALS patients may be confounded by neuropsychological impairment that could be related to FTD

#### Amyotrophic lateral sclerosis assessment questionnaires (ALSAQ-40, ALSAQ-5)

The self-reported 40 item ALSAQ-40 is a Rasch modeled instrument designed to evaluate aspects of health considered important to patients with ALS and is frequently listed as a secondary outcome in current ALS clinical trials to assess HRQoL. It was developed in accordance with FDA 2009 guidelines for PROs and covers many of the same items as the ALSFRS-R with the exception of the respiratory domain. The recall period for its five domains—communication, eating/drinking, physical mobility, activities of daily living (ADL) independence and emotional functioning, is 2 weeks and responses are on a Likert scale from 0 to 4 (never true to always true). A measure of global HRQoL impact can be obtained by summing individual domain scores for a total score between 0 (best health) to 100 (worst health) [52, 53].

To minimize patient burden, a 5-item subset of the ALSAQ-40 called the ALSAQ-5 was developed [54] with one item representing each domain. ALSAQ-40 and ALSAQ-5 scores are very highly correlated (ICC) = 0.95 at baseline, and 0.96 at follow up). Scores on the ALSAQ-5 replicated those on the ALSAQ-40 to within one or two points, suggesting the ALSAQ-5 may be a valid alternative in studies where the ALSAQ-40 is impracticable or inappropriate to use, or if HRQoL is an exploratory endpoint.

#### ALS specific quality of life—short form (ALSSQOL-SF)

The 20-item ALSSQOL-SF questionnaire measures overall QoL in individuals with ALS and is a short-form version of the original 50-item ALSSQOL-R [55]. Developed in accordance with FDA 2009 guidelines for PROs, the final items for each subscale were estimated using Modified Graded Response (MGR) analysis and addresses six domains and subscales (Negative emotion, Interaction with people and environment, Intimacy, Religiosity, Physical symptoms, and Bulbar function). Responses are on a 0–10-point scale from ‘strongly disagree’ to ‘strongly agree.’ Recall is one week, and completion time is between 2 and 4 min. The ALSSQOL-SF has 6 items that are thought to be applicable to ALS patients (pain, fatigue, excessive saliva, problems with speaking, problems with strength and ability to move, problems with sleep).

Internal consistency as measured with Cronbach’s alpha between the ALSSQOL-R and the ALSSQOL-SF ranged from 0.70 (physical symptoms) to 0.89 (religiosity). Correlation of the Physical Symptoms subscale with the ALSFRS-R was significant (r = 0.48). A comparison with ALSFRS-R subscales shows significant correlations among all, but most closely with ALSFRS-R Fine motor (r = 0.37) and ALSFRS-R Gross motor functioning (r = 0.44), and less so with ALSFRS-R Speech (r = 0.17) and Respiratory (r = 0.34) domains [55].

Although well developed and validated, the assessment of HRQoL by the ALSSQOL-SF is more applicable in a clinical setting than a clinical trial, where information about the individual patient’s overall self-perceived well-being is more useful and meaningful. When assessing the impact of a specific therapeutic intervention, global QoL instruments are likely to be insensitive, because the intervention in question targets only one of many factors affecting overall QoL; for ALS, it is physical functioning.

#### EuroQoL-5D (EQ-5D)

The EQ-5D is a 5-item, self-report measure of health status developed by the EuroQoL Group that provides a simple, generic measure of HRQoL for clinical and economic appraisal. Well validated and commonly used in clinical trials as a secondary outcome, it applies to a wide range of health conditions and treatments and provides a simple descriptive profile and a single index value for health status. It consists of 5 items across 5 domains—mobility, self-care, usual activities, pain/discomfort and anxiety/depression. HRQoL as measured by the EQ-5D visual analog scale (VAS) found worse HRQoL in ALS patients with fatigue and ventilator use during home visits [56], and in ALS patients randomized to placebo vs. oral lithium in the lithium carbonate in the amyotrophic lateral sclerosis trial (LiCALS) [57]. Patients’ HRQoL as assessed by the EQ-5D decreased with increasing severity of ALS disease with patients' mean VAS rating of their own health ranging from 0.74 for stage 1 (early) disease severity, to 0.37 for stage 4 (late stage) disease severity [58].

#### World Health Organization Quality of Life BREF Scale (WHOQOL-BREF)

The WHOQOL-BREF is a shortened version of the generic 100-item WHOQOL and was recently validated in a large ALS/MND population [59]. It consists of 26 items across 4 domains: Physical health, Psychological, Social relationships and Environment. Responses are on a Likert Scale ranging from 0 to 5 with higher scores indicating better QOL. It can provide a Total score, and independent subscores for the Physical, Psychological and Environmental domains. Reliability across the domains ranged from alpha values of 0.57 (Social) to 0.82 (Physical). Excluding the social domain, the domains on the WHOQOL-BREF showed adequate internal construct validity demonstrating invariance of age, gender and ALS onset type, and acceptable levels of unidimensionality as determined by fit to the Rasch model.

WHOQOL-BREF domains showed a significant difference and strong gradient across most ALSFRS-R levels, with the Physical domain showing significant differences between limb or bulbar onset. The total WHOQOL-BREF score was also shown to correlate with the NRS-QOL (r = 0.6493), Modified Hospital Anxiety and Depression Scale (mHADS) (r = − 0.6787), WHODAS-2.0 (World Health Organization’s Disability Assessment Schedule– 2.0) (r = − 0.6489) and EQ-5D (r = 0.6651) [60]. Further validation is required, particularly longitudinally to assess the scale’s responsiveness across time, and responsiveness to therapy in clinical trials.

#### Neuro-QoL

Neuro-QoL are brief measures of HRQoL for clinical research in neurology and quantify the physical, mental and social impacts on adults and children living with neurological conditions. Recall for Neuro-QoL measures is 7 days and response options are 5-point Likert scale (never to always; no difficulty to unable to do). In ALS patients, 1-week test–retest reliability ranged from 0.79 to 0.96 and ICCs from 0.48 (social) to 0.92 (upper extremity functioning) [24]. ALS patients who reported a worsening of their physical well-being showed significantly worse upper extremity function scores than those who reported no change (t = − 2.17), and patients who reported a decrease in overall HRQoL also showed significant worsening of upper extremity function (t = − 3.17) and a trend toward increasing fatigue (t = − 1.68) [24, 60].

To mirror ALSFRS-R subscores, the Neuro-QoL measures that assess upper extremity functioning (8 items), lower extremity functioning (8 items), speech difficulties (6 items) and swallowing difficulties (6 items) are recommended as an option to assess HRQoL in these domains. Additional Neuro-QoL measures to assess impact of ALS on fatigue (8 items) and sleep disturbance (8 items) are also recommended [24].

The Neuro-QoL Fatigue score was inversely correlated with the ALSFRS-R score. Higher fatigue correlated significantly with lower function (r = − 0.72) [24]. Ambulatory ALS patients had significantly lower NeuroQoL-fatigue scores than non-ambulatory patients [24].

#### PROMIS Global Health 10

The PROMIS Global Health has 5 physical health items (GHP) and 5 mental health items (GHM). Recall is 7 days, and response options are on a 5-point Likert scale (excellent health to poor health; ‘completely able to carry out activity’ to ‘not at all able to do activity’). The GHP and GHM scales had internal consistency reliability coefficients of 0.81 and 0.86, respectively. GPH correlated more strongly with the EQ-5D than did GMH (r = 0.74 vs. 0.56). GPH correlated most strongly with pain impact (r = − 0.75); whereas GMH correlated most strongly with depressive symptoms (r = − 0.71) [61].

PROMIS GHP and GHM scores correlated positively with the ALSFRS-R score. Lower physical and mental health correlated with lower functioning (physical: r = 0.85; mental: r = 0.58) and ambulatory ALS patients had significantly higher PROMIS-10 physical health scores than non-ambulatory patients [24].

Both Neuro-QoL and PROMIS instruments were developed following FDA 2009 Guidelines for PRO development [22]. They are well-validated, psychometrically-sound and clinically relevant measures of HRQoL and Global Health for individuals with neurological conditions such as ALS.

Table 4 provides a summary of measures to assess HRQoL and QoL in ALS.

### Cognitive screens

By end-stage disease, up to half of ALS patients will develop neuropsychological impairment [62], in some cases reaching a joint diagnosis of ALS and frontotemporal dementia (FTD). Future ALS clinical research should include cognitive screening to help provide evidence of cognitive or behavioral changes that might shorten survival, affect the assessment of ALS disease severity and progression, and potentially confound response to therapy [63]. A recent review article considered the ALS-Cognitive Behavioral Screen (ALS-CBS) and the Edinburgh cognitive and behavioral ALS screen (ECAS) to be the most suitable for detecting cognitive/behavioral changes in ALS [25]. Table 4 outlines the strengths and limitations of these two cognitive screens.

#### ALS cognitive behavior screen (ALS-CBS)

The ALS-CBS tracks the progression of cognitive/behavioral impairments in ALS. The cognitive section is clinician or care-staff administered and includes eight tasks addressing attention—concentration, tracking/monitoring, and initiation and retrieval. Scores range from 0 to 20. In general, cognitive scores from 17 to 20 do not indicate cognitive impairment. Patients with scores of 11–16 are classified as ALS cognitively impaired. Scores of 10 and below suggests the need for evaluation for ALS frontotemporal (FTD) or other dementia.

The 2-min, 15-item behavioral section rates caregiver-perceived changes in the patient since disease onset and assesses for: apathy, inhibition, empathy, emotional control, frustration tolerance, cognitive flexibility, insight, judgment, decision making, food preferences and language disturbance [64]. Items are scored from 0 to 3, with a total score ranging from 0 to 45. Scores below the cutoff (≤ 32) are classified as possible FTD-behavioral type, those scoring in the impaired range (33–36) are classified as having ALS behavior impairment, and those scoring ≥ 37 are considered ALS behaviorally normal [65].

Correlation of ALS-CBS cognitive scores was 0.7 with FVC and 0.04 with ALSFRS-R. Behavior scores correlated 0.19 with FVC and 0.08 with ALSFR-R. Compared to the gold standard of neuropsychological assessment, mean scores for both cognition and behavior of the ALS-CBS were significantly lower in ALS patients than the control group [64]. Interrater reliability for the behavior section was very high, r = 1.0 [66]. Test–retest reliability has not yet been established for this scale.

#### Edinburgh cognitive and behavioral ALS screen (ECAS)

The ECAS was launched as a rapid screening test to provide early, ALS-specific identification of cognitive and behavioral changes specific to ALS [67, 68]. The 15–20-min cognitive screen is clinician assessed while a 25-min behavioral interview is administered separately to the caregivers. The cognitive screen includes assessment of fluency, executive functions, language, memory, and visuospatial function. The domains considered specific to ALS disease are executive functions (including social cognition), language and fluency. The ECAS ALS-specific composite score ranges from 0 to 100, while the ECAS total score ranges from 0 to 136 with higher scores indicating less impairment. Reported test–retest reliability was good for the majority of subdomains (ICC > 0.70) [62]. ALS patients with bulbar involvement demonstrated significantly worse ALS-specific and total ECAS scores and impairment in behavior was significantly related to a worse ALSFRS-R score [62]. Validated against an extensive neuropsychological battery, the AUC for the ALS-specific score was 0.94 and 0.91. An ALS-specific score of ≤ 77 and an ECAS total score of ≤ 105 are the cut-off scores for “abnormality” or cognitive impairment due to ALS [69].

Both the ECAS and ALS-CBS take motor problems into consideration, but the ALS-CBS requires shorter administration time. Conversely, the ECAS assesses language and social cognition domains and might be more suitable for screening in ALS patients. Both screen for behavioral problems, which is an added advantage in this population [25].

Both these measures have been included in recent ALS clinical trials, nonetheless, additional validation would offer further insight into the scales’ test characteristics and continued usefulness in clinical trials.

## Discussion

Data generated by a PRO can provide a statement of a treatment benefit from the patient perspective. For a treatment benefit to be meaningful, there should be evidence that the PRO under consideration measures the particular concept (e.g., disease construct/attribute) that is studied. Content validity is the extent to which the content of the measure is an adequate reflection of the construct to be measured [70], and content validity is emphasized in both the EMA [71] and FDA guidelines [22] as a requirement when developing and selecting PRO measures for use in a clinical trial and potential labeling purposes. The functional measures as well as the majority of the QoL measures discussed in this paper are disease-specific measures, specific to ALS and will generally have adequate content validity if used in an ALS population similar to the ALS population that the measure was developed in. In addition, all measures have a well-defined conceptual model, and all were developed following FDA guidelines with the exception of the ALSFRS-R which was developed prior to the FDA guidelines.

The selection of appropriate endpoints for ALS clinical trials is particularly important to quantify functional status for ALS where there is no standard measure of disease progression, no single objective measure of overall disability or functional status, and a lack of widely available, well-established candidate biomarkers.

Although there is no universal “best” instrument to measure functional status, almost all ALS clinical trials to date have employed the ALSFRS-R as the primary outcome measure for assessing ALS disease progression and functional disability. There are several advantages of the ALSFRS-R that support its inclusion as a primary endpoint in ALS clinical trials—it is relatively simple, easy to administer, reliable and well-validated, cost-effective and is a proxy for survival. However, issues of non-linearity, multidimensionality and floor and ceiling effects have challenged the ALSFRS-R’s continued utility as a primary outcome measure. Rasch analysis suggests that some functional aspects are especially difficult to capture in the context of the ALSFRS-R. In response, other qualitative instruments to measure functional status in ALS including the ALSFRS-EX, ALS-MITOS, CNS-BFS, DALS-15, MND-DS, and ROADS have been developed. These newer measures could provide alternative or complementary endpoints to the ALSFRS-R, to assess functional status in ALS clinical trials. The inclusion of QOL measures and ALS cognitive screens in future clinical trials is also recommended to assess the impact of new ALS therapies more fully.

The findings from this paper demonstrate several research needs. Specifically, newer measures require additional testing and validation in future ALS clinical trials. Some of these measures are psychometrically more rigorous and sensitive than the ALSFRS-R. Using newer measures requires some willingness to move beyond the commonly used ALSFRS-R, but additional longitudinal validity data for the newer scales may pave the way for them to eventually be used as a primary outcome measure in the assessment of physical functioning in ALS trials. Further exploration of the role of digital devices and wearable technology to assess physical functioning will also play a part in the future of ALS research and with more emphasis placed on the patient experience, or the patient journey, future clinical trials research should include a measure of patient well-being such as a QoL or HRQoL. Approaches beyond functional scales as trial endpoints (e.g. time to next confirmed event) should also be further explored. Additional research also needs to continue towards establishing a simple quantitative “general use” biomarker to assess ALS and ALS progression which can then be supplemented by the qualitative measures discussed herein. Natural history studies should also be encouraged to help provide a more clearly delineated map of ALS progression and its impacts across various subgroups of patients.

An important limitation to this review was its targeted, rather than systematic approach, which may have resulted in a collection of qualitative measures that is not exhaustive of those available in the ALS field. However, we believe our supplemental search of the clinicaltrials.gov database led to the most prominent and promising measures being included herein.

## Conclusion

This paper serves as a reference guide for researchers seeking to identify potential qualitative measures of functional status for use in their ALS clinical trials. To our knowledge, this is the first paper that provides a descriptive collection of these measures including information on their strengths and weaknesses and recommendations for their implementation based on the published literature.

How to best quantify disease progression in ALS remains unclear. The measures discussed herein offer alternative or complementary options to the ALSFRS-R, in the context of the currently available tools. Additional research is needed to determine whether any of these qualitative measures of functional status, perhaps combined with a QOL measure will more accurately track and describe ALS disease progression, that could then help accelerate development of effective treatments for ALS.

## Data Availability

Not applicable.

## Abbreviations

ADL: Activities of daily living
ALS: Amyotrophic lateral sclerosis
ALSAQ: Amyotrophic Lateral Sclerosis Assessment Questionnaires
ALS-CBS: ALS-Cognitive Behavioral Screen
ALSFRS-R: Amyotrophic Lateral Sclerosis Functional Rating Scale—Revised version
ALSFRS-EX: ALS Functional Rating Scale Extension
ALS-MITOS: ALS Milano–Torino Staging
ALSSQOL-SF: ALS Specific Quality of Life—Short Form
CI: Confidence interval
CNS-BFS: Center for Neurologic Study Bulbar Function Scale
DALS-15: Dyspnea ALS Scale
ECAS: Edinburgh cognitive and behavioral ALS screen
EQ-5D: EuroQoL-5D
FDA: US Food and Drug Administration
FTD: Frontotemporal
FVC: Forced vital capacity
GHP: Global Health Physical
GHM: Global Health Mental
GRM: Grade Response Modeling
HRQoL: Health-related quality of life
ICC: Intraclass correlation
LiCALS: Lithium carbonate in the amyotrophic lateral sclerosis trial
MGD: Modified Grading Response
mHADS: Modified Hospital Anxiety and Depression Scale
MND-DS: Motor Neuron Disease—Dyspnea Scale
NIV: Non-invasive ventilation
NRS-QoL: Numerical Rating Scale—Quality of Life
PALS: People with Amyotrophic Lateral Sclerosis
PRO: Patient reported outcome
PROMIS: Patient-Reported Outcomes Measurement Information System
QoL: Quality of life
ROADS: Rasch Overall ALS Disability Scale
ROC AUC: Receiver operating curve area under the curve
VC: Vital capacity
WHODAS-2.0: World Health Organization’s Disability Assessment Schedule—2.0
